# Chimeric H5 influenza virus-like particle vaccine elicits broader cross-clade antibody responses in chickens than in ducks

**DOI:** 10.3389/fvets.2023.1158233

**Published:** 2023-06-15

**Authors:** Jaekeun Park, Chang-Seon Song, David Hyunjung Chung, Sangyong Choi, Junghoon Kwon, Sungsu Youk, Dong-Hun Lee

**Affiliations:** ^1^College of Veterinary Medicine, Konkuk University, Seoul, South Korea; ^2^Department of Veterinary Medicine, Virginia-Maryland College of Veterinary Medicine, University of Maryland, College Park, MD, United States; ^3^Department of Pathobiology and Veterinary Sciences, University of Connecticut, Storrs, CT, United States; ^4^Department of Nutritional Sciences, University of Connecticut, Storrs, CT, United States; ^5^College of Veterinary Medicine, Kyungpook National University, Daegu, Republic of Korea; ^6^College of Medicine and Medical Research Institute, Chungbuk National University, Cheongju, Republic of Korea

**Keywords:** highly pathogenic avian influenza virus, vaccine, virus like particle, insect cell, poultry, chicken, duck

## Abstract

Eurasian-lineage highly pathogenic avian influenza (HPAI) H5 viruses have spread throughout Asia, the Middle East, Europe, Africa, and most recently, North and South America. These viruses are independently evolving into genetically and antigenically divergent clades, and broad-spectrum vaccines protecting against these divergent clades are needed. In this study, we developed a chimeric virus-like particle (VLP) vaccine co-expressing hemagglutinins from two clades (clades 1 and 2.3.2.1) of HPAI H5 viruses and performed comparative cross-clade hemagglutination inhibition (HI) analysis in chickens and ducks. The chimeric VLP immunization induced a significantly broader spectrum of antibodies against various clades of HPAI H5 viruses than monovalent VLPs both in chickens and ducks. While the chimeric VLP led to broadened antibody responses in both species, significantly lower levels of HI antibodies were elicited in ducks than in chickens. Moreover, boost immunization failed to increase antibody responses in ducks regardless of the VLPs used, in contrast to chickens that showed significantly enhanced antibody responses upon boost immunization. These results suggest (1) the potential application of the chimeric VLP technology in poultry to help control HPAI H5 viruses by offering broader antibody responses against antigenically different strains and (2) possible obstacles in generating high levels of antibody responses against HPAI H5 viruses in ducks via vaccination, implying the need for advanced vaccination strategies for ducks.

## 1. Introduction

Highly pathogenic avian influenza (HPAI) viruses cause high mortality in Gallinaceous bird species. Since 1996, the Eurasian-origin A/goose/Guangdong/1/1996 (Gs/GD) lineage of HPAI H5Nx has caused outbreaks in poultry and wild birds around the world except for Antarctica and Australia (1). The Gs/GD lineage HPAI virus has evolved independently into 10 genetically and antigenically distinct clades (from clades 0 to 9) and their subclades (2, 3), making it difficult to control them with vaccination in poultry. While the clade 2 viruses have widely spread and predominated in poultry residing in most parts of the world since 2005, the clade 1 viruses remained predominant in Southeast Asia (4, 5). Moreover, continuous human infections with the Gs/GD lineage HPAI H5 viruses in enzootic regions have often been fatal and raised concerns about potential human pandemics (6, 7).

To reduce economic losses in the poultry industry and the risk of human infection, vaccination of domestic poultry against HPAI H5 has been extensively utilized in various enzootic regions, including China, Egypt, Indonesia, and Vietnam (8). However, current vaccines have a critical limitation in that they cannot elicit broad-spectrum antibody responses against diverse clades of HPAI H5 viruses (9). Antigenic match between the vaccine strain and the locally circulating virus is thus a critical factor in achieving optimal vaccine efficacy and controlling the spread of HPAI H5 viruses. Indeed, an outbreak of antigenically distinct HPAI H5 strains in vaccinated poultry was reported to cause significant morbidity and mortality despite of vaccination (10). The decrease in vaccine efficacy is also experimentally demonstrated in heterologous challenge studies using chickens (11, 12) and domestic ducks (11, 13), necessitating the development of vaccines offering broad protection against different clades of H5 viruses.

Domestic ducks play a critical role in maintaining and transmitting HPAI H5 viruses to various host species, including poultry and wild birds (14, 15). Given their significant contribution to the epidemiology of HPAI, it is imperative to mitigate the risk of virus infection in domestic ducks through vaccination in order to control the spread of HPAI. Antigenically-matching vaccination has proven effective in protecting domestic ducks against infection and clinical signs following a homologous HPAI H5 virus challenge (16). Currently developed vaccines, however, have shown suboptimal protective efficacy in domestic ducks, allowing morbidity, mortality, and prolonged viral shedding upon challenges with antigenically distant HPAI H5 viruses (11, 13). Despite its importance, there has been a lack of development in vaccines aimed at providing broader immunity to domestic ducks against antigenically distant clades of HPAI H5 viruses. Virus-like particles (VLPs), which resemble infectious virus particles in structure and morphology, have been suggested as the new generation of vaccines against various viruses, including various influenza A viruses (17–20). In particular, chimeric influenza VLPs containing hemagglutinins (HAs) derived from multiple subtypes of influenza viruses were shown to provide protection from multiple subtypes of influenza viruses in ferrets (21). Recently, Kang et al. also demonstrated the protective efficacy of chimeric influenza VLPs expressing HAs of clade 2.3.2.1c and clade 2.3.4.4cHPAI H5 viruses in chickens (22). However, the potential of chimeric influenza VLPs has not been demonstrated in domestic ducks.

In this study, we generated chimeric VLPs simultaneously expressing antigenically remote HAs from clades 1 and 2 HPAI H5 viruses using the baculovirus expression vector system (BEVS). Our objective was to investigate the enhanced antibody responses elicited by the chimeric VLPs against four antigenically distant clades of HPAI H5N1 viruses. We conducted our investigations in two major poultry species: chickens and domestic ducks.

## 2. Materials and methods

### 2.1. Generation of recombinant baculoviruses

**Figure 1:**
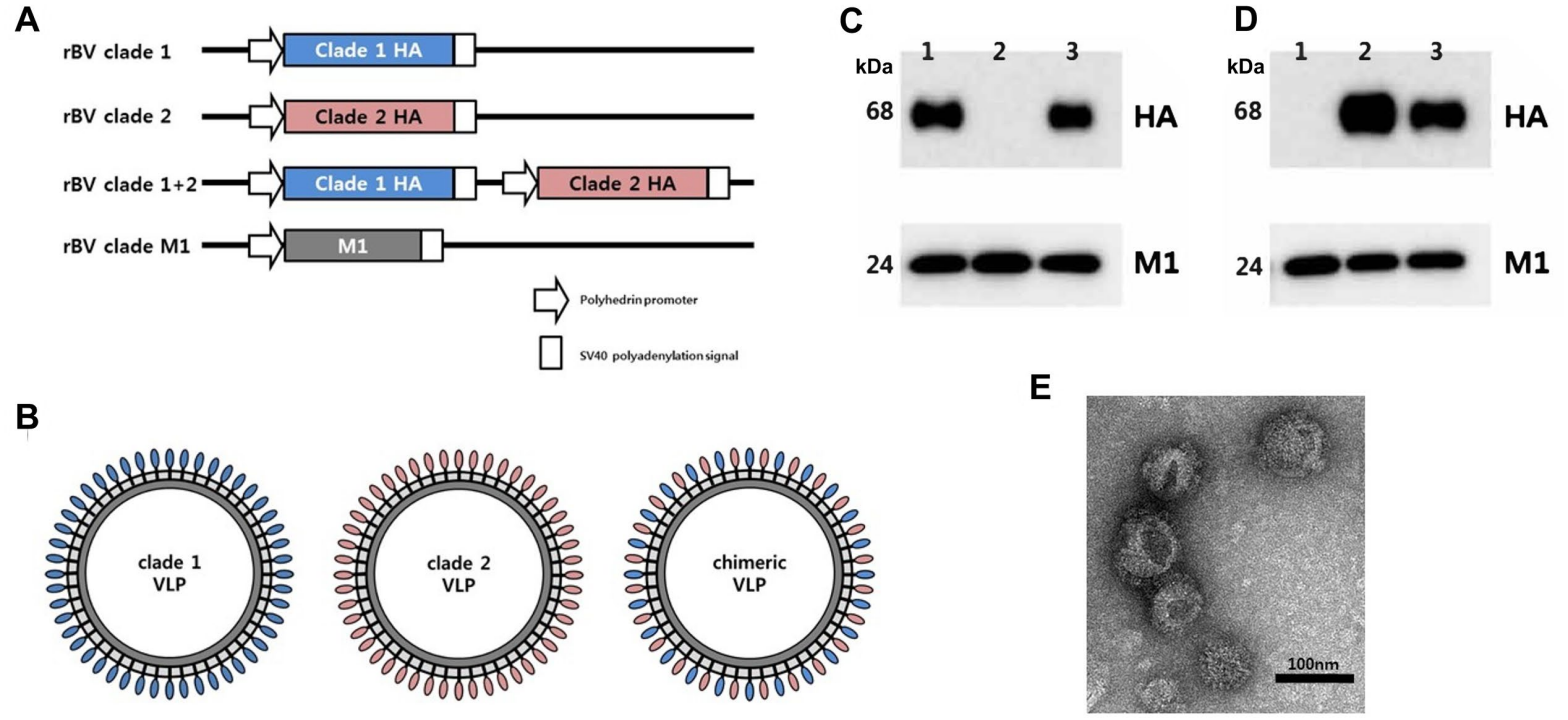
Preparation and characterization of chimeric H5 VLP vaccine containing hemagglutinin (HA) proteins from different clades of highly pathogenic avian influenza (HPAI) H5 viruses. **(A)** Recombinant baculoviruses (rBV) encoding clade 1 HA, clade 2 HA, or both clade 1 HA and clade 2 HA were used to infect Sf9 cells. Sf9 cells were co-infected with rBV encoding HA gene and rBV encoding influenza matrix 1 (M1) gene to generate **(B)** clade 1, clade 2, or clade 1 + 2 VLPs. Four micrograms of clade 1 VLP (lane 1), clade 2 VLP (lane 2), and chimeric VLP (lane 3) were characterized by Western blotting using **(C)** anti-clade 1 HA monoclonal antibody with anti-influenza M1 antibody or **(D)** anti-clade 2 monoclonal antibody with anti-influenza M1 antibody. HA and M1 are expected to be 68 kDa and 28 kDa, respectively. **(E)** Transmission electron microscope was used to take images of negative staining for the chimeric VLPs. Scale bar represents 100 nm.

For cloning the full-length HA gene of clade 1 H5N1 virus, the HA gene of A/Vietnam/1194/2004 (H5N1) virus was chemically synthesized (Bioneer, Republic of Korea) without the multi-basic cleavage site (MBCS) sequence. For cloning the full-length HA gene of clade 2 H5N1 virus, viral RNA was extracted from A/mandarin duck/K10-483/2010 (H5N1, clade 2.3.2.1), the HA gene was amplified (23), and the MBCS was removed as previously described (24). The amplified HA gene from clade 1 or clade 2 virus was cloned into the vector pFastBac1 (Thermo Fisher, United States), and the resulting plasmids were designated as pFast_clade1 and pFast_clade2, respectively. The other pFastBac1 simultaneously containing both HA genes of clade 1 and clade 2 H5N1 viruses was constructed by cloning a SnaBI/HpaI-digested fragment from pFast_clade 2 into the HpaI-digested site of pFast_clade 1, and the resulting plasmid was designated as pFast_clade1 + 2. A plasmid containing an influenza matrix1 (M1) gene, designated as pFast_M1, was constructed by cloning the full-length M1 gene of A/Puerto Rico/8/1934 (H1N1) into the empty vector pFastBac1 (25). Using pFast_clade 1, pFast_clade 2, pFast_clade 1 + 2, and pFast_M1, recombinant baculovirus (rBV) encoding clade 1 HA gene, clade 2 HA gene, clade 1 and clade 2 HA genes, or influenza M1 gene was generated using a Bac-to-Bac BEVS (Thermo Fisher), and the resulting rBVs were designated as rBV_clade 1, rBV_clade 2, rBV_clade 1 + 2, or rBV_M1, respectively (Figure 1A). The titers of rBVs were measured by standard plaque assay using *Spodoptera frugiperda* (Sf9) insect cells.

### 2.2. Production and characterization of H5 VLPs and preparation of VLPs vaccines

For clade 1 H5 VLPs production, Sf9 cells were co-infected with rBV_clade1 and rBV_M1, both at a multiplicity of infection (MOI) of 5. For clade 2 H5 VLPs production, Sf9 cells were co-infected with rBV_clade2 and rBV_M1, both at an MOI of 5. For chimeric H5 VLPs production, Sf9 cells were co-infected with rBV_clade 1 + 2 and rBV_M1, both at an MOI of 5. After 72 h of infection, the culture medium containing VLPs (Figure 1B) was collected and clarified by low-speed centrifugation (2,000 × g, 30 min, 4°C) to remove large cell debris, and VLPs from the clarified supernatants were pelleted (30,000 × g, 1.5 h, 4°C). The pellet was resuspended in phosphate-buffered saline (PBS; pH 7.2), loaded onto a 20–50% (w/v) discontinuous sucrose density gradient, and ultra-centrifuged (150,000 × g, 1.5 h, 4°C) for purification. The bands positioned above the 50% sucrose density were collected. The total protein concentration of each H5 VLPs preparation was quantified using the Bradford protein assay kit (Pierce, United States) according to the manufacturer’s instructions. Expression of the clade 1 and 2 HA as well as M1 proteins in each VLPs preparation was detected by Western blotting using mouse anti-clade 1 H5 monoclonal antibody (Median Diagnostics, Republic of Korea), mouse anti-clade 2 H5 monoclonal antibody (Bionote, Republic of Korea), and rabbit anti-M1 polyclonal antibodies (Immune Technology, United States), followed by incubation with horseradish peroxidase (HRP)-conjugated goat anti-mouse or anti-rabbit IgG (AbD Serotec, United Kingdom). Four micrograms of each VLPs preparation were used per well for Western blotting. The presence of VLPs was observed by transmission electron microscopy (TEM; Tecnai G2 Spirit, FEI, Netherlands, installed at Korea Basic Science Institute) using a negative staining method. The H5 VLPs vaccines with final concentrations of 40 μg of VLPs/0.5 mL·dose were prepared by emulsifying each H5 VLPs collection in the oil adjuvant Montanide ISA70 (SEPPIC, France) at a ratio of 30:70 (v/v).

### 2.3. Immunization of animals and determination of serological immune responses

A total of 30 6-week-old SPF white leghorn chickens (Namduck Sanitec, Republic of Korea) and 30 5-week-old commercially available Pekin ducks (kindly provided by the Moran Food & Breeding Company, Republic of Korea) were divided into respective 3 groups (10 chickens per group and 9–10 ducks per group). Each group of chickens or ducks was intramuscularly immunized twice (three weeks apart) with clade 1, clade 2, or chimeric H5 VLPs vaccines (0.5 mL per animal). All the animals were confirmed for seronegativity before immunization using the hemagglutination inhibition (HI) test against different clades of H5N1 viruses as described below.

To determine the seronegativity of the animals before immunization and seroconversion after immunization, sera were collected from VLPs-vaccinated chickens and ducks before immunization and at 3 weeks after each immunization for cross-clade HI test using H5N1 viruses from different clades. H5N1 viruses containing HA genes, without MBCS sequences, of A/Vietnam/1194/2004 (clade 1), A/Indonesia/5/2005 (clade 2.1), A/mandarin duck/K10-483/2010 (clade 2.3.2.1), or A/chicken/Korea/ES/2003 (clade 2.5) were generated using reverse genetics (RG) system as previously described (26). HI tests were performed according to the OIE standard method using 4 HA units of H5N1 viruses. To eliminate non-specific HI factors, 1 volume of duck serum was treated with 3 volumes of receptor-destroying enzyme (Denka Seiken Co., JAPAN) at 37°C for 16 h followed by heat inactivation for 30 min at 56°C.

### 2.4. Statistical analysis

Dunn’s multiple comparison test was used following Kruskal–Wallis test (non-parametric one-way ANOVA) to compare HI titers between groups (i.e., clade 1 VLPs vs. clade 2 VLPs vs. chimeric VLPs). A two-tailed non-parametric Mann–Whitney test was used to compare HI titers between two groups (i.e., prime vs. boost or chicken vs. duck). An HI titer of 2 was assigned to samples with undetected HI activity for statistical analyses. Log-transformed (base 2) HI titers were used for statistical analysis. Results with *p*-values <0.05 were considered statistically significant.

### 2.5. Ethics statement

All animal procedures performed in this study were reviewed and approved by the Institutional Animal Care and Use Committee (IACUC) of Konkuk University.

## 3. Results

### 3.1. Generation and characterization of chimeric H5 VLPs

The chimeric H5 VLPs containing both clade 1 and clade 2 HA were observed to be released into the culture supernatants (Figures 1C–E). Western blotting analysis showed the presence of both clade 1 (Figure 1C) and clade 2 (Figure 1D) H5 HAs from the chimeric VLP, while standard VLPs only possessed either clade 1 HA or clade 2 HA as expected. Matrix proteins were detected at comparable levels between VLPs (Figures 1C,D). The size of VLP particles was approximately 100 nm in diameter, while the morphology resembled influenza virion with spikes on the surface (Figure 1E). These results confirm the successful generation of the chimeric VLPs.

### 3.2. Antibody responses in chickens

**Figure 2:**
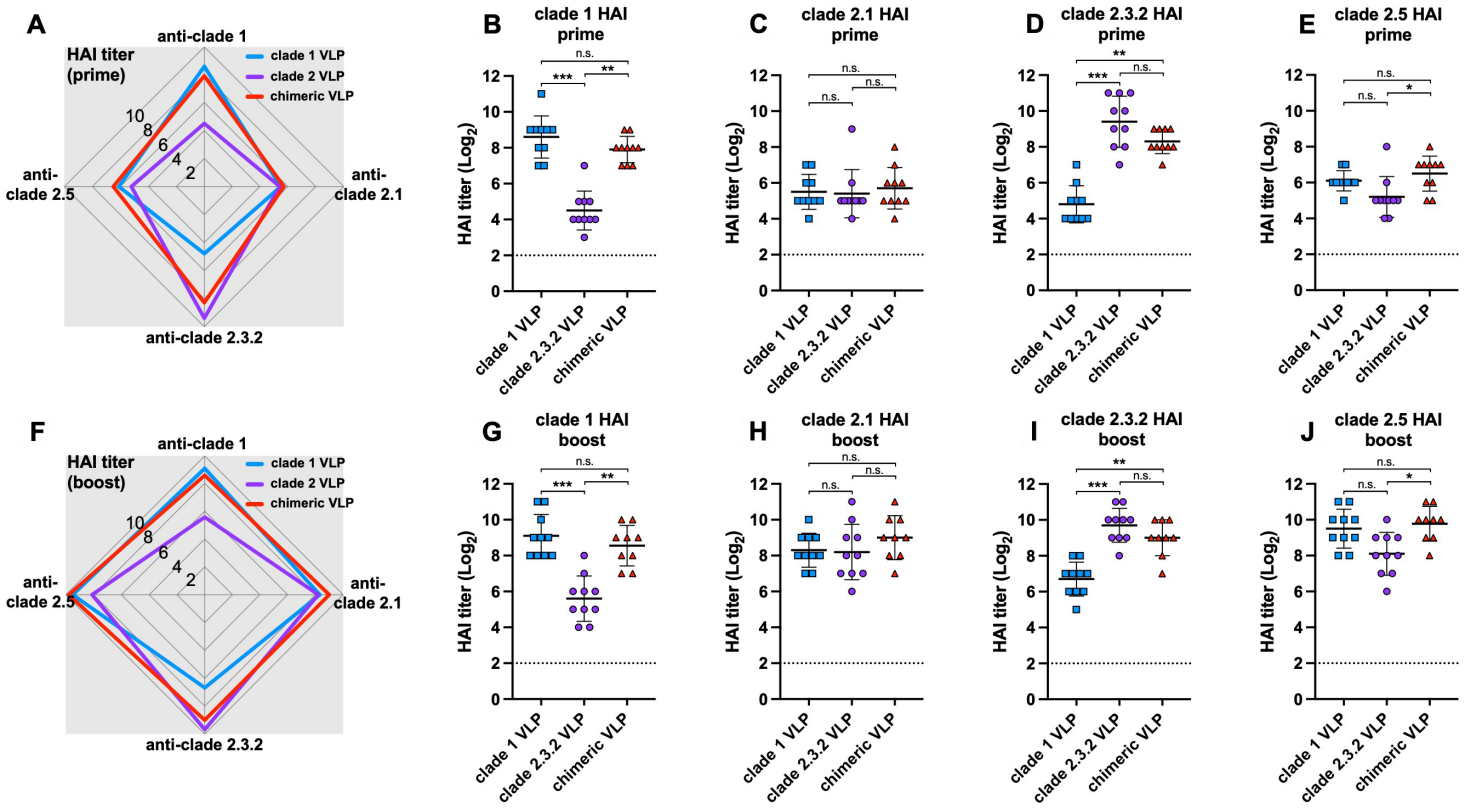
Antibody responses against chimeric H5 VLP vaccines in chickens. 6-week-old SPF chickens (*n* = 10 per group) were immunized with clade 1, clade 2, or chimeric H5 VLP vaccines (40 μg of VLP/0.5 mL·dose). Serum HI titers against different clades of HPAI H5N1 viruses were determined using antigenically different H5N1 viruses 3 weeks after priming **(A–E)** and boosting **(F–J)**. Radar charts show geometric mean serum HI titers (log2) from chickens immunized with clade 1 (blue), clade 2 (purple), or chimeric (red) H5 VLP vaccines against clade 1, 2.1, 2.3.2.1, and 2.5 H5N1 viruses. Each symbol in dot plots represents an individual animal. Horizontal lines and error bars in dot plots represent geometric mean serum HI titers and standard deviations, respectively. Dashed lines show the detection limit of the HAI assay. An HI titer of 2 was assigned to samples with undetected HI activity for generating graphs and statistical analyses. Dunn’s multiple comparison test was used following Kruskal–Wallis test (non-parametric one-way ANOVA) to compare HI titers between groups (*p < 0.05, **p < 0.01, ***p < 0.001, n.s. = not significant).

The chimeric VLPs elicited broader antibody responses against multiple HPAI H5 viruses from different clades compared to the monovalent VLPs (Figures 2A,F). Both prime and boost immunization of chimeric VLPs induced significantly higher HI antibodies against clade 1 (Figures 2B,G) and clade 2.3.2 (Figures 2D,I) H5N1 viruses compared to each standard VLPs. For example, the levels of anti-clade 1 antibodies elicited by chimeric VLPs were significantly higher than those induced by the monovalent clade 2.3.2 VLPs and comparable to those induced by monovalent clade 1 VLPs (Figures 2B,G). While the antibody response was moderate against the clade 2.1 (Figures 2C,H) and clade 2.5 viruses (Figures 2E,J) which are different from the HA clades incorporated in the chimeric VLPs, the chimeric VLP induced significantly higher anti-clade 2.5 antibodies compared to that induced by monovalent clade 2.3.2 VLP both after prime and boost immunization. These data show that the chimeric VLPs induce broader antibody responses against multiple HPAI H5 viruses from various clades compared to the monovalent VLPs.

### 3.3. Antibody responses in ducks

**Figure 3:**
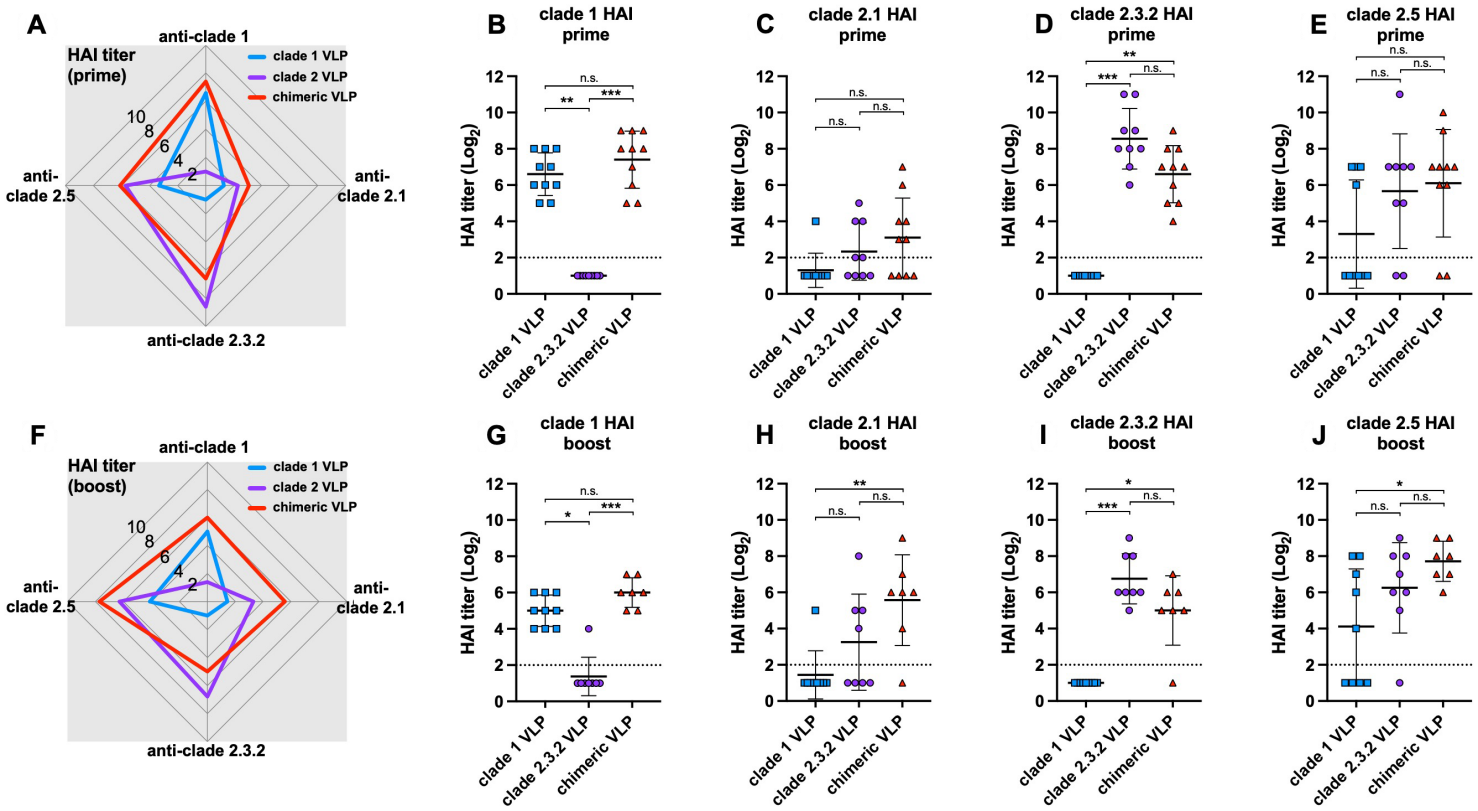
Antibody responses against chimeric H5 VLP vaccines in ducks. 5-week-old commercial ducks (n = 9–10 per group) were immunized with clade 1, clade 2, or chimeric H5 VLP vaccines (40 μg of VLP/0.5 mL·dose). Serum HI titers against different clades of HPAI H5N1 viruses were determined using antigenically different H5N1 viruses 3 weeks after priming **(A–E)** and boosting **(F–J)**. Radar charts show geometric mean serum HI titers (log2) from chickens immunized with clade 1 (blue), clade 2 (purple), or chimeric (red) H5 VLP vaccines against clade 1, 2.1, 2.3.2.1, and 2.5 H5N1 viruses. Each symbol in dot plots represents an individual animal. Horizontal lines and error bars in dot plots represent geometric mean serum HI titers and standard deviations, respectively. Dashed lines show the detection limit of the HAI assay. An HI titer of 2 was assigned to samples with undetected HI activity for generating graphs and statistical analyses. Dunn’s multiple comparison test was used following Kruskal–Wallis test (non-parametric one-way ANOVA) to compare HI titers between groups (*p < 0.05, **p < 0.01, ***p < 0.001, n.s. = not significant).

Similar to the results from chickens, the chimeric VLPs elicited broader antibody responses in ducks against multiple HPAI H5 viruses compared to the monovalent VLPs (Figures 3A,F). After both prime and boost immunization, the chimeric VLPs induced significantly higher HI antibody titers against clade 1 (Figures 3B,G) and clade 2.3.2 (Figures 3D,I) H5N1 viruses compared to each monovalent VLPs. For example, the levels of anti-clade 1 antibodies elicited by the chimeric VLPs were significantly higher than those induced by the monovalent clade 2.3.2 VLPs and comparable to those induced by the monovalent clade 1 VLPs (Figures 3B,G). The chimeric VLPs induced higher mean HI antibody titers against clade 2.1 (Figures 3C,H) and clade 2.5 (Figures 3E,J) H5N1 viruses compared to each monovalent VLPs. Particularly, the chimeric VLPs induced significantly higher anti-clade 2.1 as well as anti-clade 2.5 antibodies compared to the monovalent clade 1 VLPs after the boost immunization (Figures 3H,J). These data suggest that the chimeric VLPs can induce broader antibody responses compared to the monovalent VLPs in ducks, in correspondence with the results from chickens. However, the induction of cross-reactive antibodies against heterologous HAs (i.e., clade 2.1 and clade 2.5) was lower than what was observed in chickens.

### 3.4. Comparison of antibody responses in chickens and ducks

**Figure 4:**
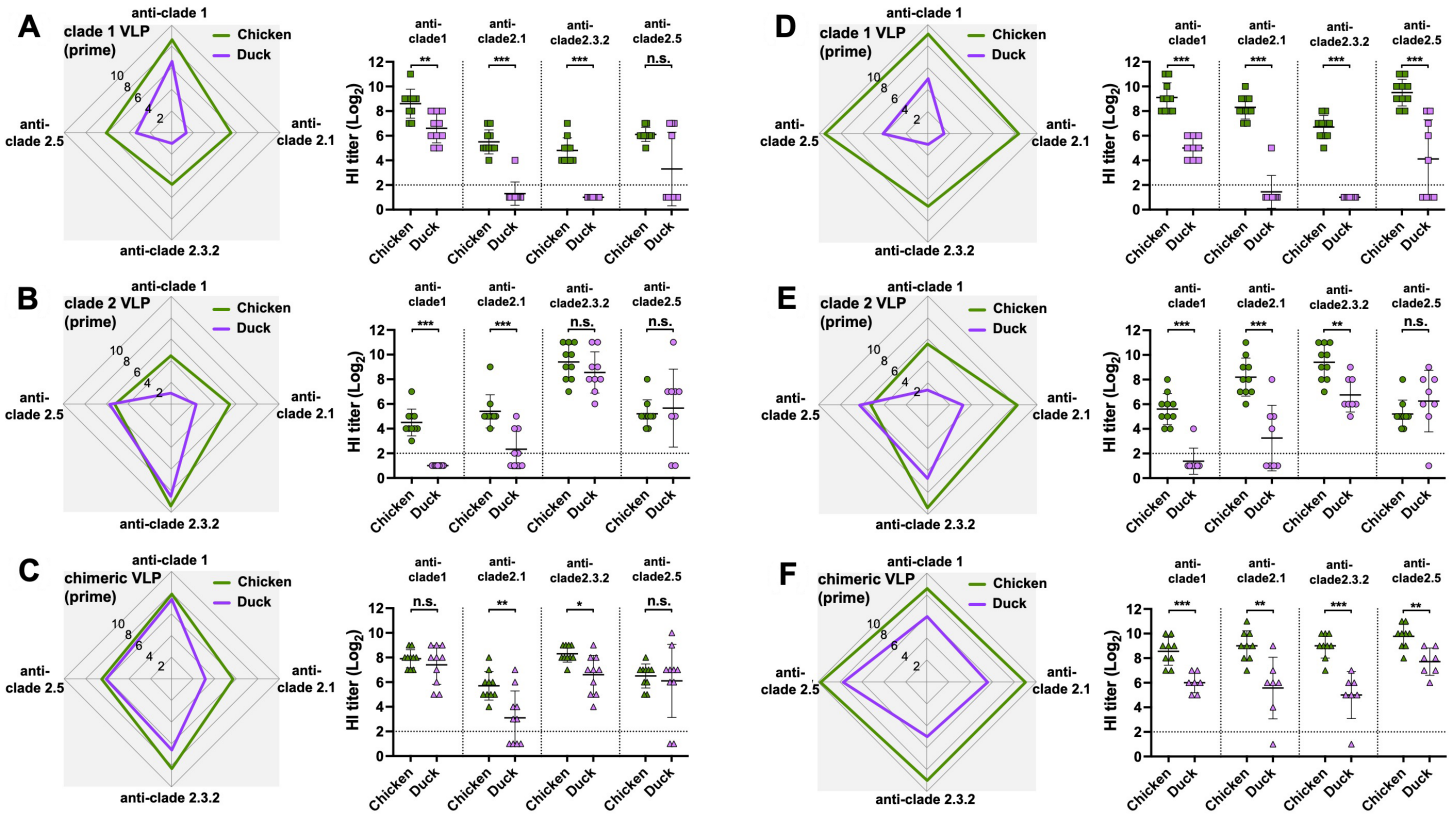
Comparison of the effect of VLP immunization on serum HI titers between chickens and ducks. The effect of VLP immunization on serum HI titers were compared between chickens and ducks using data from Figures 2, 3. The HI titers elicited by **(A)** clade 1, **(B)** clade 2, or **(C)** chimeric H5 VLP vaccines (40 μg of VLP/0.5 mL·dose) in SPF chickens (green) and commercial ducks (purple) were compared 3-weeks after priming. Similarly, the HI titers elicited by **(D)** clade 1, **(E)** clade 2, or **(F)** chimeric H5 VLP vaccines (40 μg of VLP/0.5 mL·dose) in SPF chickens (green) and commercial ducks (purple) were compared 3-weeks after boosting. HI titers against clade 1, 2.1, 2.3.2.1, and 2.5 H5N1 viruses were measured. Horizontal lines and error bars in dot plots represent geometric mean serum HI titers and standard deviations, respectively. Dashed lines show the detection limit of the HAI assay. An HI titer of 2 was assigned to samples with undetected HI activity for generating graphs and statistical analyses. A two-tailed nonparametric Mann–Whitney test was used for the comparison (*p < 0.05, **p < 0.01, ***p < 0.001, n.s. = not significant).

**Figure 5:**
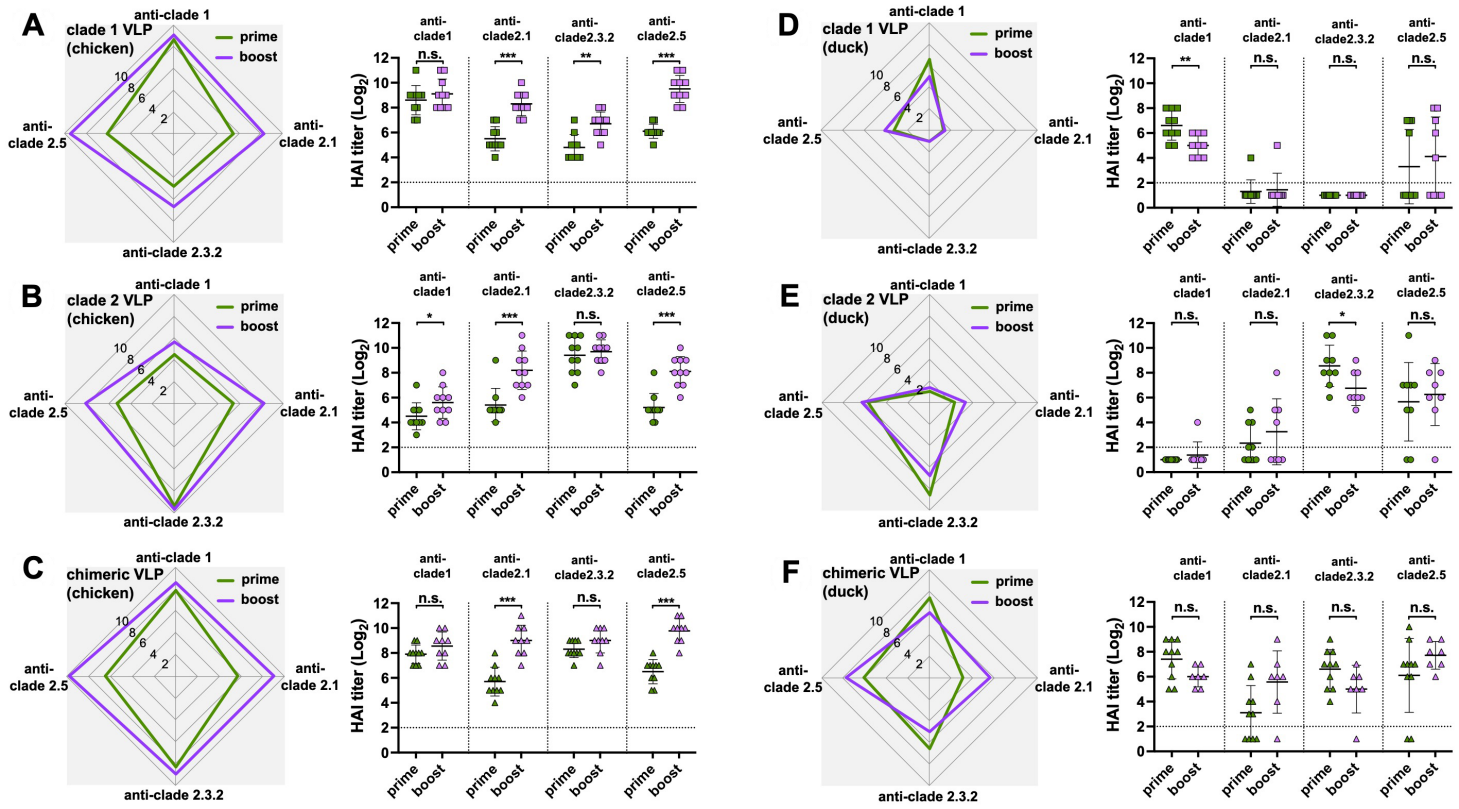
Boost-immunization significantly increased antibody responses in chickens but not in ducks. The effect of boost immunization on serum HI titers in chickens and ducks were investigated using data from Figures 2, 3. The boosting effect of **(A)** clade 1, **(B)** clade 2, or **(C)** chimeric H5 VLP vaccines (40 μg of VLP/0.5 mL·dose) in SPF chickens was investigated by comparing the serum HI titers between the primed and boosted serum HI titers against clade 1, 2.1, 2.3.2.1, and 2.5 H5N1 viruses. Similarly, the boosting effect of **(D)** clade 1, **(E)** clade 2, or **(F)** chimeric H5 VLP vaccines (40 μg of VLP/0.5 mL·dose) in commercial ducks was investigated by comparing the serum HI titers against different H5N1 viruses. Horizontal lines and error bars in dot plots represent geometric mean serum HI titers and standard deviations, respectively. Dashed lines show the detection limit of the HAI assay. An HI titer of 2 was assigned to samples with undetected HI activity for generating graphs and statistical analyses. A two-tailed nonparametric Mann–Whitney test was used for the comparison (*p < 0.05, **p < 0.01, ***p < 0.001, n.s. = not significant).

The antibody responses in ducks were significantly narrower and lower than those in chickens throughout the study, regardless of the VLPs used and the number of immunizations. Except for the HI antibodies against the clade 2.5 HA, ducks generated significantly lower levels of antibody responses than chickens following the prime immunization with either monovalent clade 1 or clade 2 VLPs (Figures 4A,B) and the chimeric VLPs (Figure 4C). The differences were even greater following the boost immunization. Across all four clades of HAs, ducks generated significantly lower levels of antibody responses compared to chickens regardless of the VLPs used (Figures 4D–F). While boost immunization significantly broadened cross-clade antibody responses in chickens (Figures 5A–C), this effect was not observed in ducks which did not show any increase in antibody responses upon the boost immunization (Figures 5D–F).

## 4. Discussion

In this study, we developed the chimeric influenza VLP vaccine containing HAs from two different clades of HPAI H5 viruses and performed a comparative evaluation of the vaccine’s potential uses in chickens and ducks against the globally circulating Eurasian-lineage H5 HPAI viruses. Our chimeric H5 VLP vaccine incorporating HAs from clades 1 and 2 HPAI H5N1 viruses induced broader HI antibodies than monovalent VLPs in chickens and domestic ducks, indicating the potential for a broad protective efficacy against different clades of HPAI H5 viruses. We also showed that antibody responses to the VLPs in ducks were significantly narrower and lower than those in chickens, regardless of the type of antigens used and the number of immunizations, suggesting that more advanced vaccine or vaccination strategies would be required to elicit broad and robust antibody responses in ducks.

Although this study successfully demonstrated the potential of chimeric VLP technology to broaden antibody responses to multiple clades of HPAI H5 viruses in chickens and ducks, there are several limitations. Firstly, our comparison of antibody breadth between chickens and ducks was based solely on HI assays. Although HI titers generally correlate well with neutralizing titers and are widely accepted as indicators of influenza protection (27, 28), it is important to consider that different species may exhibit varying levels of neutralizing antibodies despite similar HI titers. While this study did not include neutralization assays, it is possible that such assays could provide higher sensitivity in assessing the level of protective antibodies in immunized animals and serve as better predictors of the broadened protective efficacy of the chimeric VLPs compared to HI assays. Secondly, the lack of challenge experiments in this study prevents us from determining whether the relatively low titers of HI antibodies in ducks would result in lower levels of protection against HPAI H5 viruses in ducks compared to chickens. Protection from viral infections is not solely determined by pre-existing immunity but is also influenced by host anti-viral responses and the susceptibility of hosts to different viral strains, which are factors that evolutionarily differentiate chickens and ducks. For example, RIG-I in ducks was shown to play a suppressive role in viral replication and initiate pro-inflammatory pathways involving type I interferon signals at the sites of infection and was suggested as one of the key pathways causing the differences in susceptibilities to avian influenza viruses between chickens and ducks (29). Therefore, it remains uncertain whether the generally lower HI titers following VLP immunization in ducks relative to chickens would result in lower levels of protection in ducks. In fact, studies by Webster et al. have demonstrated complete protection from HPAI H5N1 virus challenges in vaccinated ducks with HI titers much lower than those observed in chickens (30), indicating that low HI antibody titers may not necessarily predict a lack of protection against HPAI viruses in domestic ducks. Ultimately, it is crucial to conduct challenge studies in both chickens and ducks, using multiple clades of HPAI H5 viruses, to confirm the broadened protective efficacy of the chimeric VLPs and to determine whether the lower HI titers in ducks correspond to a decrease in protective efficacy.

Both current and previous studies have demonstrated the chimeric VLPs as a promising vaccine platform to provoke enhanced protective efficacy against antigenically distant influenza viruses in chickens (22). While the effectiveness of chimeric VLPs may vary across poultry species against HPAI, the chimeric VLP vaccine platform remains a promising approach for controlling HPAI epidemics. Future studies will include comparative viral challenge experiments in order to gain further insights into broadly-protective vaccination, host species immunity, and the varying pathogenic mechanisms of AIVs in different poultry species.

The chimeric VLP vaccine developed in this study demonstrated the induction of a high level of broadly reacting HI antibodies against various H5 viruses in immunized chickens and similar but relatively lower responses in ducks. These findings suggest that the chimeric VLP technology holds promise as a platform for controlling the Gs/GD-lineage HPAI H5 viruses in poultry. In addition, since the VLPs have only HA and M1 proteins, it will allow differentiation of virus-infected birds from vaccinated birds by detecting antibodies to viral nucleocapsid, as we showed in our previous studies (17–19), which offers a promising strategy for differentiating infected from vaccinated animals.

## Data availability statement

The raw data supporting the conclusions of this article will be made available by the authors, without undue reservation.

## Ethics statement

The animal study was reviewed and approved by Institutional Animal Care and Use Committee (IACUC) of Konkuk University.

## Author contributions

D-HL and C-SS: conceptualization and funding acquisition. D-HL and JP: methodology. JK, SY, D-HL, and JP: formal analysis. JK, SY, DC, JP, and SC: investigation. DC, JP, and SC: data curation. JP and D-HL: writing—original draft preparation. JK, SY, C-SS, DC, and SC: writing—review and editing. All authors have read and agreed to the published version of the manuscript.

## Funding

D-HL is supported by Korea Institute of Planning and Evaluation for Technology in Food, Agriculture and Forestry (IPET) through Animal Disease Management Technology Development Program, funded by Ministry of Agriculture, Food and Rural Affairs (MAFRA) (grant number: 122057–2).

## Conflict of interest

The authors declare that the research was conducted in the absence of any commercial or financial relationships that could be construed as a potential conflict of interest.

## Publisher’s note

All claims expressed in this article are solely those of the authors and do not necessarily represent those of their affiliated organizations, or those of the publisher, the editors and the reviewers. Any product that may be evaluated in this article, or claim that may be made by its manufacturer, is not guaranteed or endorsed by the publisher.

## References

[1] Lee DH Bertran K Kwon JH Swayne DE. Evolution, global spread, and pathogenicity of highly pathogenic avian influenza H5Nx clade 2.3.4.4. J Vet Sci. (2017) 18:269–08. doi: 10.4142/jvs.2017.18.S1.269, PMID: 28859267 PMC5583414

[2] Sonnberg S Webby RJ Webster RG. Natural history of highly pathogenic avian influenza H5N1. Virus Res. (2013) 178:63–77. doi: 10.1016/j.virusres.2013.05.009, PMID: 23735535 PMC3787969

[3] Donis RO Smith GJ. Nomenclature updates resulting from the evolution of avian influenza a(H5) virus clades 2.1.3.2a, 2.2.1, and 2.3.4 during 2013-2014. Influenza Other Respir Viruses. (2015) 9:271–6. doi: 10.1111/irv.12324, PMID: 25966311 PMC4548997

[4] Thor SW Nguyen H Balish A Hoang AN Gustin KM Nhung PT. Detection and characterization of clade 1 Reassortant H5N1 viruses isolated from human cases in Vietnam during 2013. PLoS One. (2015) 10:e0133867. doi: 10.1371/journal.pone.0133867, PMID: 26244768 PMC4526568

[5] Le TH Nguyen NT. Evolutionary dynamics of highly pathogenic avian influenza a/H5N1 HA clades and vaccine implementation in Vietnam. Clin Exp Vacc Res. (2014) 3:117–7. doi: 10.7774/cevr.2014.3.2.117, PMID: 25003084 PMC4083063

[6] Beigel JH Farrar J Han AM Hayden FG Hyer R de Jong MD. Avian influenza a (H5N1) infection in humans. N Engl J Med. (2005) 353:1374–85. doi: 10.1056/NEJMra052211, PMID: 16192482

[7] Webby RJ Webster RG. Are we ready for pandemic influenza? Science. (2003) 302:1519–22. doi: 10.1126/science.1090350, PMID: 14645836

[8] Swayne DE Spackman E Pantin-Jackwood M. Success factors for avian influenza vaccine use in poultry and potential impact at the wild bird-agricultural interface. EcoHealth. (2014) 11:94–8. doi: 10.1007/s10393-013-0861-3, PMID: 24026475

[9] Criado MF Sá e Silva M Lee DH Salge CAL Spackman E Donis R. Cross-protection by inactivated H5 Prepandemic vaccine seed strains against diverse goose/Guangdong lineage H5N1 highly pathogenic avian influenza viruses. J Virol. (2020) 94:e00720-20. doi: 10.1128/JVI.00720-20, PMID: 32999029 PMC7925181

[10] Salaheldin AH Veits J Abd el-Hamid HS Harder TC Devrishov D Mettenleiter TC. Isolation and genetic characterization of a novel 2.2.1.2a H5N1 virus from a vaccinated meat-turkeys flock in Egypt. Virol J. (2017) 14:48. doi: 10.1186/s12985-017-0697-5, PMID: 28274236 PMC5343302

[11] Pfeiffer J Suarez DL Sarmento L To TL Nguyen T Pantin-Jackwood MJ. Efficacy of commercial vaccines in protecting chickens and ducks against H5N1 highly pathogenic avian influenza viruses from Vietnam. Avian Dis. (2010) 54:262–1. doi: 10.1637/8715-031909-Reg.1, PMID: 20521643

[12] Kapczynski DR Pantin-Jackwood MJ Spackman E Chrzastek K Suarez DL Swayne DE. Homologous and heterologous antigenic matched vaccines containing different H5 hemagglutinins provide variable protection of chickens from the 2014 U.S. H5N8 and H5N2 clade 2.3.4.4 highly pathogenic avian influenza viruses. Vaccine. (2017) 35:6345–53. doi: 10.1016/j.vaccine.2017.04.042, PMID: 28456525

[13] Cha RM Smith D Shepherd E Davis CT Donis R Nguyen T. Suboptimal protection against H5N1 highly pathogenic avian influenza viruses from Vietnam in ducks vaccinated with commercial poultry vaccines. Vaccine. (2013) 31:4953–60. doi: 10.1016/j.vaccine.2013.08.046, PMID: 23994373

[14] Gilbert M Chaitaweesub P Parakamawongsa T Premashthira S Tiensin T Kalpravidh W. Free-grazing ducks and highly pathogenic avian influenza. Thailand Emerg Infect Dis. (2006) 12:227–4. doi: 10.3201/eid1202.050640, PMID: 16494747 PMC3373083

[15] Kwon JH Bahl J Swayne DE Lee YN Lee YJ Song CS. Domestic ducks play a major role in the maintenance and spread of H5N8 highly pathogenic avian influenza viruses in South Korea. Transbound Emerg Dis. (2020) 67:844–1. doi: 10.1111/tbed.13406, PMID: 31675474

[16] Pantin-Jackwood MJ DeJesus E Costa-Hurtado M Smith D Chrzastek K Kapczynski DR. Efficacy of two licensed avian influenza H5 vaccines against challenge with a 2015 U.S. H5N2 clade 2.3.4.4 highly pathogenic avian influenza virus in domestic ducks. Avian Dis. (2019) 63:90–6. doi: 10.1637/11895-050918-Reg.1, PMID: 31251524

[17] Lee DH Park JK Lee YN Song JM Kang SM Lee JB. H9N2 avian influenza virus-like particle vaccine provides protective immunity and a strategy for the differentiation of infected from vaccinated animals. Vaccine. (2011) 29:4003–7. doi: 10.1016/j.vaccine.2011.03.067, PMID: 21463681 PMC5555295

[18] Lee DH Park JK Song CS. Progress and hurdles in the development of influenza virus-like particle vaccines for veterinary use. Clin Exp Vac Res. (2014) 3:133–9. doi: 10.7774/cevr.2014.3.2.133, PMID: 25003086 PMC4083065

[19] Park JK Lee DH Youn HN Kim MS Lee YN Yuk SS. Protective efficacy of crude virus-like particle vaccine against HPAI H5N1 in chickens and its application on DIVA strategy. Influenza Other Respir Viruses. (2013) 7:340–8. doi: 10.1111/j.1750-2659.2012.00396.x, PMID: 22716302 PMC4941755

[20] Roldao A Mellado MC Castilho LR Carrondo MJ Alves PM. Virus-like particles in vaccine development. Expert Rev Vaccines. (2010) 9:1149–76. doi: 10.1586/erv.10.115 20923267

[21] Pushko P Pearce MB Ahmad A Tretyakova I Smith G Belser JA. Influenza virus-like particle can accommodate multiple subtypes of hemagglutinin and protect from multiple influenza types and subtypes. Vaccine. (2011) 29:5911–8. doi: 10.1016/j.vaccine.2011.06.068, PMID: 21723354

[22] Kang YM Cho HK Kim JH Lee SJ Park SJ Kim DY. Single dose of multi-clade virus-like particle vaccine protects chickens against clade 2.3.2.1 and clade 2.3.4.4 highly pathogenic avian influenza viruses. Sci Rep. (2021) 11:13786. doi: 10.1038/s41598-021-93060-8, PMID: 34215796 PMC8253753

[23] Hoffmann E Stech J Guan Y Webster RG Perez DR. Universal primer set for the full-length amplification of all influenza a viruses. Arch Virol. (2001) 146:2275–89. doi: 10.1007/s007050170002, PMID: 11811679

[24] Heckman KL Pease LR. Gene splicing and mutagenesis by PCR-driven overlap extension. Nat Protoc. (2007) 2:924–2. doi: 10.1038/nprot.2007.132, PMID: 17446874

[25] Park JK Lee DH Yuk SS Tseren-Ochir EO Kwon JH Noh JY. Virus-like particle vaccine confers protection against a lethal Newcastle disease virus challenge in chickens and allows a strategy of differentiating infected from vaccinated animals. Clin. Vac. Immunol. (2014) 21:360–5. doi: 10.1128/CVI.00636-13, PMID: 24403523 PMC3957659

[26] Lee DH Park JK Kwon JH Yuk SS Erdene-Ochir TO Jang YH. Efficacy of single dose of a bivalent vaccine containing inactivated Newcastle disease virus and reassortant highly pathogenic avian influenza H5N1 virus against lethal HPAI and NDV infection in chickens. PLoS One. (2013) 8:e58186. doi: 10.1371/journal.pone.0058186, PMID: 23469269 PMC3585801

[27] Molesti E Wright E Terregino C Rahman R Cattoli G Temperton NJ. Multiplex evaluation of influenza neutralizing antibodies with potential applicability to in-field serological studies. J Immunol Res. (2014) 2014:457932:1–11. doi: 10.1155/2014/457932, PMID: 25101305 PMC4101955

[28] Benne CA Kroon FP Harmsen M Tavares L Kraaijeveld CA de Jong JC. Comparison of neutralizing and hemagglutination-inhibiting antibody responses to influenza a virus vaccination of human immunodeficiency virus-infected individuals. Clin Diagn Lab Immunol. (1998) 5:114–7. doi: 10.1128/CDLI.5.1.114-117.1998, PMID: 9455891 PMC121402

[29] Evseev D Magor KE. Innate immune responses to avian influenza viruses in ducks and chickens. Vet Sci. (2019) 6. doi: 10.3390/vetsci6010005, PMID: 30634569 PMC6466002

[30] Webster RG Webby RJ Hoffmann E Rodenberg J Kumar M Chu HJ. The immunogenicity and efficacy against H5N1 challenge of reverse genetics-derived H5N3 influenza vaccine in ducks and chickens. Virology. (2006) 351:303–11. doi: 10.1016/j.virol.2006.01.044, PMID: 16690097

